# Optimal Condition to Create Femoral Tunnel Considering Combined Influence of Knee Flexion and Transverse Drill Angle in Anatomical Single-Bundle ACL Reconstruction Using Medial Portal Technique: 3D Simulation Study

**DOI:** 10.1155/2018/2643247

**Published:** 2018-07-17

**Authors:** Sung-Hwan Kim, Sung-Jae Kim, Chong Hyuk Choi, Dohyun Kim, Min Jung

**Affiliations:** The Arthroscopy and Joint Research Institute, The Department of Orthopaedic Surgery, Yonsei University College of Medicine, Seoul, Republic of Korea

## Abstract

There has been no previous study using three-dimensional (3D) measurement on femoral tunnel characteristics according to the combined influence of various flexion angles of knee and transverse drill angles in single-bundle ACL reconstruction with transportal technique. The purpose of this study was to determine optimal condition of knee flexion angle and transverse drill angle to create secure femoral tunnel in single-bundle ACL reconstruction with transportal technique considering tunnel length, tunnel wall breakage, and graft bending angle. This study was conducted using simulation of 3D computed tomography of thirty subjects. Three variables of femoral tunnel changed according to combined influence of four flexion angles of knee and three transverse drill angles were measured: tunnel length, wall breakage, and graft bending angle. There was no case of short femoral tunnel less than 25 mm at 120° and 130° of flexion. There was no case of breakage of femoral tunnel at 120° of flexion with maximum transverse drill angle (MTA) and MTA-10° and at 130° of flexion. Considering effect on graft bending angle, decrease of flexion angle and transverse drill angle could be appropriate in creating femoral tunnel. Increased flexion angle and transverse drill angle secured femoral tunnel having sufficiently long length without wall breakage. However, avoiding excessive flexion angle and maximum transverse drill angle could be recommended because they tended to cause more acute graft bending angle.

## 1. Introduction

In performing ACL reconstruction, the placement of graft tunnel has been recognized as one of the most important factors to obtain satisfactory postoperative outcomes. In particular, femoral tunnel placement was turned out to be crucial because it has a great influence on knee kinematics [1, 2]. In the 80s and 90s, although considered to be less anatomic, femoral tunnel was created mostly using transtibial technique. However, in transtibial technique, there is a constraint in creating femoral tunnel by tibial tunnel and the femoral tunnel shows a tendency to be located at a higher position compared to native ACL footprint. Biomechanical studies revealed that this drawback of transtibial technique led to deficiency in restoring the normal knee joint kinematics including rotational stability [3–5], and clinical studies also showed poor outcomes with regard to rotational stability [6]. To compensate these shortcomings, efforts were made to position the femoral tunnel at the anatomical position of native ACL and anatomical ACL reconstruction using transportal technique [7, 8] or outside-in technique [9, 10] was presented as alternatives. Transportal technique has an advantage of no need for lateral femoral dissection using an additional incision necessary for outside-in technique. However, the femoral tunnel created with transportal technique through medial portal has shortcomings such as short tunnel length [11–13], posterior wall blowout [12], and cartilage damage [14, 15]. In this respect, previous studies insisted that flexion angle should be increased more than 90° [16, 17], and medial portal should be made at lower position [18] to avoid unsatisfactory femoral tunnel. A lower flexion angle causes a shorter femoral tunnel, and transverse drill angle through medial portal made at more lateral position leads to posterior wall blowout [18]. The transverse drill angle also affects the trajectory of femoral tunnel [19]. However, these studies did not present the optimal condition in consideration of the femoral tunnel characteristics changing according to the combined effect of knee flexion angle and transverse drill angle. Additionally, the angular change of the graft at the femoral tunnel aperture depending on the condition to create tunnel was not taken into account. A previous study demonstrated that the increased acuity of the graft bending angle at the femoral tunnel aperture led to increased bending stress on the graft [20]. Repetitive motion of the reconstructed graft at the sharp edge due to the acute femoral tunnel angle could result in injury of graft and tunnel widening [20, 21]. It was noted that femoral graft bending angle in ACL reconstruction with transportal technique was more acute than angle in ACL reconstruction with transtibial technique [22]. Accordingly, it is necessary to take an integrated approach to the proper condition to create femoral tunnel, considering femoral tunnel length, tunnel wall breakage, and graft bending angle comprehensively. To the best of the authors' knowledge, there has been no previous study using three-dimensional (3D) measurement on the femoral tunnel characteristics including tunnel length, tunnel wall breakage, and graft bending angle, which changed according to the combined influence of various flexion angles of knee and transverse drill angles in single-bundle ACL reconstruction with transportal technique. The purpose of the present study was to determine the optimal condition of the knee flexion angle and transverse drill angle to create femoral tunnel in single-bundle ACL reconstruction with use of transportal technique considering tunnel length, tunnel wall breakage, and graft bending angle. It was hypothesized that there would be an appropriate knee flexion angle and transverse drill angle for creation of optimal femoral tunnel. This study was conducted using 3D computed tomography (CT) simulation.

## 2. Materials and Methods

### 2.1. Reconstruction of 3D Computed Tomographic Model

CT images of knees of subjects who had a CT scan to evaluate trauma around the knee from August 2009 to March 2011 were retrospectively reviewed after approval by the institutional review board of our institution. The CT scanner Sensation 64 (Siemens Healthcare, Erlangen, Germany) was used for all examinations. The tube parameters were 120 kVp and 135~253 mAs. The acquisition matrix was 512 x 512 pixels. The scan field of view was 134 ~ 271 mm, and the slice thickness was 0.6~1 mm. A CT scan was conducted in full extension. The subjects who met the following criteria were included in the present study: (1) no ligament injury, (2) no osseous deformity, (3) no fracture of femur and tibia, (4) no operation history, and (5) grade 0 or I according to the Kellgren-Lawrence grading scale [23]. CT images of thirty subjects were included. Digital Imaging and Communications in Medicine (DICOM) data were extracted from the picture archiving and communication system (Centricity PACS, GE Medical System Information Technologies, Milwaukee, Wisconsin). The coronal, sagittal, and axial images were imported into Mimics software (version 17, Materialise, Leuven, Belgium) and 3D bone model of knee including femur and tibia without soft tissue was reconstructed.

### 2.2. 3D Simulation of Femoral Tunnel Drilling

**Figure 1:**
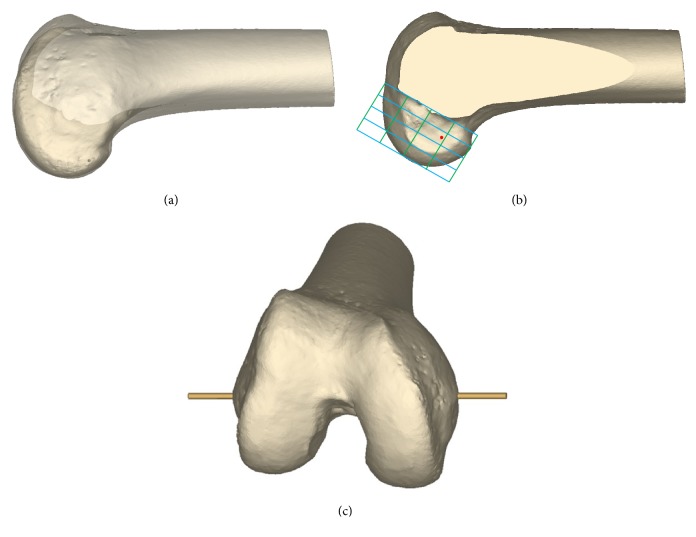
(a) 3D reconstructed model of femur was aligned in a true lateral position, where medial and lateral femoral condyles were superimposed. (b) The medial femoral condyle was virtually eliminated from the original 3D model at the most anterior aspect of the intercondylar notch, and A 4 × 4 grid was drawn on the medial wall of the lateral femoral condyle from a true medial view of the femur established at 90° of knee flexion. The femoral footprint center was determined according to the following coordinates: The distance parallel to the Blumensaat line was 28.4% along the line measured from the posterior border. The distance perpendicular to the Blumensaat line was 35.7% along the line measured from the Blumensaat line. (c) Transepicondylar axis was established as rotation axis of flexion to make change of the flexion angle.

The femoral footprint center was determined using method described by Forsythe et al. using 3D-reconstructed model [24]. To establish femoral center of ACL footprint, 3D reconstructed model of femur was aligned in a true lateral position, where medial and lateral femoral condyles were superimposed as the position for the quadrant method developed by Bernard et al. (Figure 1(a)) [25]. And then the medial femoral condyle was virtually eliminated from the original 3D model at the most anterior aspect of the intercondylar notch to have a visual on the medial wall of the lateral femoral condyle (Figure 1(b)) [26]. A 4 × 4 grid was drawn on the medial wall of the lateral femoral condyle from a true medial view of the femur established at 90° of knee flexion, similar to the quadrant method for standard lateral radiograph previously described (Figure 1(b)) [25]. The most anterior edge of the intercondylar notch was used as the reference for the grid alignment in place of Blumensaat line on the standard lateral radiograph. The femoral footprint center was determined according to the coordinates of the reference point noted by the previous study [27] on the single-bundle anatomic reconstruction using the quadrant method. The coordinates were defined by the segments of the grid along the Blumensaat line and perpendicular to the Blumensaat line. The distance of the femoral center parallel to the Blumensaat line was 28.4% along the line measured from the posterior border. The distance perpendicular to the Blumensaat line was 35.7% along the line measured from the Blumensaat line. The point was displayed at the determined position as the femoral footprint center (Figure 1(b)). And then the original 3D femur model including both condyles was restored.

**Figure 2:**
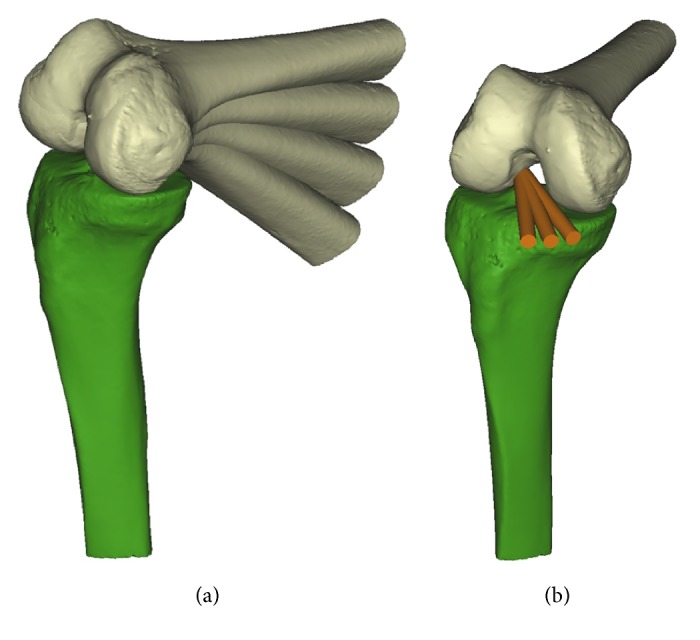
Variables at the twelve conditions by combination of flexion angle and transverse drill angle were measured. (a) The knee flexion angles were changed at intervals of 10° from 100° to 130° on the transepicondylar axis. (b) The possible maximum transverse drill angle of rotation can be achieved without coming into contact with the cartilage of medial femoral condyle. The first drill angle was determined as the maximum transverse drill angle (MTA) and the other two drill angles were determined as MTA-10° and MTA-20° by moving drill laterally.

To make change of the flexion angle, transepicondylar axis was used as rotation axis of flexion [28, 29] (Figure 1(c)). On the transepicondylar axis, the knee flexion angles were changed at intervals of 10° from 100° to 130° (Figure 2(a)). 90° of flexion angle was excluded from the study because the femoral tunnel created at the 90° of flexion angle was reported to have a tendency to blow out the posterior cortex of the lateral femoral condyle [16]. The transverse drill angle was set in three positions. According to the previous study [19], the possible maximum transverse drill angle of rotation can be achieved without coming into contact with the cartilage of medial femoral condyle. The first drill angle was determined as the maximum transverse drill angle (MTA) and the other two drill angles were determined as MTA-10° and MTA-20° by moving drill laterally (Figure 2(b)). During the actual operation, femoral tunnel can be created through the far anteromedial portal while viewing through the anterolateral portal. This allows these two portals to be freely positioned and the far anteromedial portal to be positioned at MTA, MTA-10°, and MTA-20°. The drill bit was simplified to the virtual cylinder. The cylinder passed through the center of the virtual far anteromedial portal and the determined femoral footprint center. During the actual operation of ACL reconstruction using medial portal technique, the position of far anteromedial portal should be as low as possible just above the medial meniscus without damaging the anterior horn of the medial meniscus. In the present study with 3D-reconstructed model, the center of the virtual far anteromedial portal was established at 10 mm above the tibial plateau in consideration of the radius of the femoral tunnel and the thickness of the medial meniscus. The diameter of drill bit was set to 8 mm.

### 2.3. Measurement of Variables of the Femoral Tunnel Characteristics

**Figure 3:**
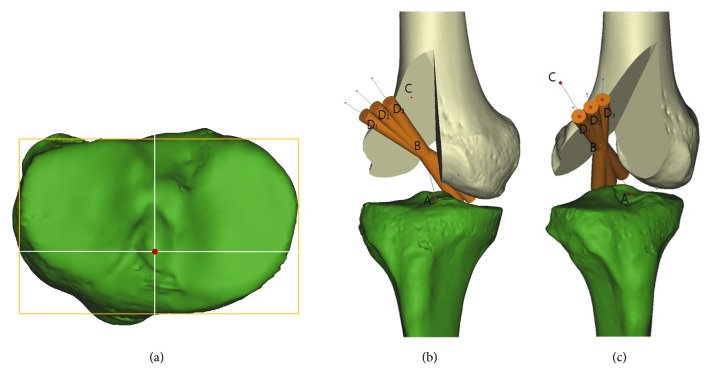
The graft bending angle at the femoral tunnel aperture was measured as the angle between the femoral tunnel penetrated by virtual cylinder and the extended line that passed through the tibial footprint center and the femoral footprint center with the knee in full extension. (a) The tibial footprint center was determined according to the following coordinates: the point located at 35.7% of the anterior-to-posterior depth of the tibia measured from the anterior border and at 51.5% of the medial-to-lateral width of the tibia measured from the medial border. (b) Anterior view. (c) Oblique view. Point A is the tibial footprint center, and point B is the femoral footprint center. Extended line that passed through these two centers is the line connecting points A, B, and C. Point D indicates the center of femoral tunnel at the external cortex. The graft bending angle is measured as the angle composed of points C, B, and D.

Three major variables changed according to the combined influence of four flexion angles of knee and three transverse drill angles were measured: (1) femoral tunnel length, (2) femoral tunnel wall breakage, and (3) graft bending angle at the femoral tunnel aperture. Each variable at the twelve conditions by combination of flexion angle and transverse drill angle was measured. Maximum femoral tunnel length was determined as the distance from the intra-articular center of femoral footprint to the center of the external cortex of the lateral femoral condyle penetrated by the virtual cylinder. A minimum length of the femoral tunnel without wall breakage for the secure graft fixation was set as 25 mm based on previous research [16]. The tunnel with less than 25 mm of length was regarded as a short tunnel. Comparison of tunnel lengths between four different knee flexion angles in condition of fixed transverse drill angle and between three different transverse drill angles in condition of fixed knee flexion angle were performed. The femoral tunnel wall breakage was divided into two types of entrance breakage and mid-tunnel breakage. Entrance breakage meant the breakage of wall at the entrance of the tunnel, and mid-tunnel breakage meant the breakage of wall within 25 mm from the entrance of the tunnel without entrance breakage. The graft bending angle at the femoral tunnel aperture was measured as the angle between the femoral tunnel penetrated by virtual cylinder and the extended line that passed through the tibial footprint center and the femoral footprint center (Figure 3) [30]. The tibial footprint center was determined according to the coordinates of the reference point noted by the previous study [31] using 3D reconstructed model [24]. On a true proximal-to-distal view of the tibial plateau, the point that was located at 35.7% of the anterior-to posterior depth of the tibia measured from the anterior border and at 51.5% of the medial-to-lateral width of the tibia measured from the medial border was set as the tibial footprint center (Figure 3). According to a previous study [32], consistent contact pressure occurred at the anterior aspect of the tunnel and anterior portion of the graft had maximum contact pressure with the knee in full extension. Therefore, in the present study, the graft bending angle at the femoral tunnel aperture was measured with the knee in full extension.

### 2.4. Statistical Analysis

The repeated-measures Analysis of Variance (ANOVA) for continuous variables was performed to compare the tunnel lengths and the graft bending angles. The post hoc analysis with adjusted P-value obtained by Bonferroni correction was performed to make pairwise comparisons. Cochran's Q test for dichotomous variables was performed to compare the proportions of short tunnel and tunnel breakage between groups. The level of significance was set at P<0.05. Statistical analysis was conducted using the IBM SPSS Statistics for Windows software program (version 23.0; IBM, Armonk, New York).

## 3. Results

The demographic data of patients were listed in Table 5. Comparison of tunnel lengths between different flexion angles of 100° to 130° showed statistically significant difference in condition of transverse drill angle fixed at MTA, MTA-10°, or MTA-20° (P<0.001) (Table 1). Post hoc analysis showed that there were statistically significant differences (P<0.05) in all pairwise comparisons except a comparison between 120° and 130° of flexions at MTA-10° (P=0.158) and MTA-20° (P=0.516) (Table 6). In condition of flexion angle fixed at 100°, 110°, 120°, or 130°, comparison of tunnel lengths between different transverse drill angles of MTA to MTA-20° showed a statistically significant difference (P<0.001) (Table 1). Post hoc analysis showed that there were statistically significant differences in all pairwise comparisons (P<0.001) (Table 7).

In terms of short tunnel, at 100° of flexion, the proportion of short tunnel with less than 25 mm was 63.3% (19 cases) at MTA, 90.0% (27 cases) at MTA-10°, and 100% (30 cases) at MTA-20°. At 110° of flexion, the proportion of short tunnel with less than 25 mm was 10.0% (3 cases) at MTA, 6.7% (2 cases) at MTA-10°, and 6.7% (2 cases) at MTA-20°. There was no short tunnel at 120° and 130° of flexion. The proportion of short tunnel according to the different transverse drill angle differed significantly at only 100° of flexion (P<0.001). The proportion of short tunnel according to the different flexion angle of knee differed significantly at all transverse drill angles (P<0.001) (Table 2).

Regarding femoral tunnel wall breakage, there were more than one case of breakage at 100° and 110° of flexion regardless of transverse drill angle and 120° of flexion and MTA-20°. The femoral tunnel created at 120° of flexion and MTA, MTA-10°, and 130° of flexion had no breakage of wall (Table 8). The proportion of femoral tunnel wall breakage according to the different transverse drill angle differed significantly at 100°, 110°, and 120° of flexion (P≤0.001). The proportion of femoral tunnel wall breakage according to the different flexion angle of knee differed significantly at all transverse drill angles (P<0.001) (Table 3).

According to the results of comparison of graft bending angles at femoral tunnel aperture between each setting, in condition of transverse drill angle fixed at MTA, MTA-10°, or MTA-20°, graft bending angles between different flexion angles of 100° to 130° had significant difference (P<0.001) (Table 4). Post hoc analysis showed that there were statistically significant differences in all pairwise comparisons (P<0.05) (Table 9). As knee flexion angle increased, graft bending angle also increased. In condition of flexion angle fixed at 100°, 110°, 120°, or 130°, comparison of graft bending angles between different transverse drill angles of MTA to MTA-20° showed a statistically significant difference (P<0.05) (Table 4). Post hoc analysis showed that there were statistically significant differences in all pairwise comparisons (P<0.05) (Table 10). As transverse drill angle increased by moving the drill medially in close proximity to the cartilage of medial femoral condyle, graft bending angle also increased.

## 4. Discussion

The technical difficulties of creating femoral tunnel in ACL reconstruction with use of transportal technique through medial portal are attributed to the occurrence risk for short tunnel length [11–13], posterior wall blowout [12], and cartilage damage [14, 15]. The characteristics of femoral tunnel were influenced by flexion angle of knee [16, 17, 19] and transverse drill angle [19]. Therefore, combined effect of flexion angle of knee and transverse drill angle on creation of femoral tunnel has an important clinical relevance, and these two factors should be considered comprehensively not individually. The present study focused on the optimal condition of the knee flexion angle and transverse drill angle to create femoral tunnel considering tunnel length, tunnel wall breakage, and graft bending angle in single-bundle ACL reconstruction using transportal technique.

According to the results of present study, both flexion angle of knee and transverse drill angle had significant effects on the length of femoral tunnel. In condition of each fixed transverse drill angle, as flexion angle increased, most of tunnel lengths tended to increase with statistical significance. To attain a high flexion angle up to 130° was beneficial to achieve a longer femoral tunnel. Basdekis et al. [16] also reported that the femoral tunnel length was longer at 110° and 130° of flexion than at 90° of flexion in their cadaveric study on ACL reconstruction using transportal technique. In condition of each fixed flexion angle of knee, as transverse drill angle decreased by moving the drill laterally, tunnel length tended to decrease at 100° of flexion and increase at more than 100° of flexion. In making femoral tunnel at 100° flexion angle, femoral tunnel tended to pass through the low position of femoral condyle. Therefore, transverse drill angle by the more lateral position tended to break the posterior condyle, leading to short tunnel length. When the tunnel less than 25 mm in length was regarded as a short tunnel, there was no case of a short tunnel in creating at 120° and 130° of flexion. Only considering the tunnel length, high flexion angle of 120° and 130° could be optimal condition, and longer tunnel could be achieved by decreased transverse drill angle moving the drill medially.

In terms of tunnel wall breakage, there was no case of entrance breakage and mid-tunnel breakage at 120° of flexion with MTA and MTA-10° and 130° of flexion. There were one or more cases of tunnel wall breakage at 100°, 110°, and 120° of flexion with MTA-20°. As flexion angle increased and transverse drill angle increased by moving drill medially, the proportion of tunnel wall breakage tended to decrease. A previous study [16] measured the closest distance between guide pin for femoral tunnel and the posterior cortex. The distance was -0.5±2.1 mm at 90° of flexion, 5.1±1.1 mm at 110° of flexion, and 9.9±2.5 mm at 130° of flexion. Both this previous study [16] and the present study showed that as flexion angle increased, the risk of tunnel wall breakage decreased. There was no case of breakage at 130° of flexion regardless of transverse drill angle. However, at 120° of flexion, there was not a case of breakage at MTA and MTA-10° but seven cases of breakage at MTA-20°. As transverse drill angle decreased, the femoral tunnel length increased, but the problem was that the risk of wall breakage also increased. Accordingly, it requires the attention and effort to increase the transverse drill angle by moving drill medially at less than 130° of flexion. Considering two factors of length and wall breakage of femoral tunnel, it could be recommended to create femoral tunnel at increased flexion angle and increased transverse drill angle for a secure femoral tunnel having sufficiently long length without wall breakage.

However, if the influence of the femoral graft bending angle was applied, the optimal condition in creating femoral tunnel changed. There has been no study on change of graft bending angle according to the combined effect of flexion angle and transverse drill angle in single-bundle ACL reconstruction using transportal technique. As knee flexion angle increased, graft bending angle also increased. The increased acuity of the graft bending angle at the femoral tunnel aperture was reported to cause increased bending stress on the graft, leading to injury of the graft and tunnel widening [20, 21]. A previous study [16] dealing with effect of only flexion angle presented the results of two-dimensional measurement on X-ray using eight cadaveric specimens. It proposed that 110° of flexion angle was more optimum condition compared to 90° and 130° of flexion angle [16]. The present study also demonstrated that it would be better to avoid too high flexion angle and less than 130° of flexion could be more appropriate. In addition to the flexion angle of knee, transverse drill was also considered in the present study. Increase of transverse drill angle caused graft bending angle to increase at all flexion angles of knee. Accordingly, considering the effect on graft bending angle, decrease of flexion angle and decrease of transverse drill angle could be appropriate in creating femoral tunnel. All factors considered, 120° of flexion angle of knee provided sufficient tunnel length without tunnel wall breakage, and MTA-10° led to the more decreased graft bending angle compared to MTA. Consequently, 120° of flexion angle and MTA-10° could be recommended as the optimal condition in creating femoral tunnel with sufficient tunnel length, no breakage of tunnel wall, and decreased graft bending angle at femoral tunnel aperture.

There were several limitations that warrant review before definitive conclusions can be drawn. First, the present study was conducted by simulation using 3D reconstructed CT model. Biomechanics of knee joint during flexion such as femoral roll back and screw home movement were not considered. Simplified cylinder created virtually replaced femoral tunnel. The data were obtained from virtual measurements. Further actual clinical study on real patients is needed to add the clinical significance to the present study. Second, only 3D bone model of knee including femur and tibia was reconstructed with CT images. When positioning a virtual far anteromedial portal, the thickness of the medial meniscus was considered. However, soft tissues around knee joint such as collateral ligament, cruciate ligament, and meniscus were not reconstructed. Third, the femoral and tibial centers of ACL footprint were determined according to previous studies [27, 31]. However, there are considerable variations of the ACL footprint anatomy between individuals. There is an avoidable limitation in applying the results of the current study uniformly to all patients. Fourth, measured variables of the present study were tunnel length, tunnel wall breakage, and graft bending angle. However, there are more significant variables related to the characteristics of femoral tunnel including aperture morphology [19, 33]. An integrated study including various factors not addressed in this study is needed to reach a solid conclusion.

## 5. Conclusions

In creating femoral tunnel in ACL reconstruction with transportal technique, characteristic factors including tunnel length, tunnel wall breakage, and graft bending angle should be considered comprehensively. Increased flexion angle and transverse drill angle secured femoral tunnel having sufficiently long length without wall breakage. However, avoiding excessive flexion angle and maximum transverse drill angle could be recommended because they tended to cause more acute graft bending angle. The present study helps to understand the combined effect of influential factors on the characteristics of femoral tunnel in ACL reconstruction.

## Figures and Tables

**Table 1:** Comparison of mean femoral tunnel lengths in each condition according to the flexion angle and transverse drill angle.

Flexion angle	Transverse drill angle			
	MTA	MTA-10°	MTA-20°	P-value^a^
100° (mm)	23.5±3.0 (16.1~28.0)	19.9±4.1 (12.0~28.1)	15.2±3.7 (8.6~21.9)	<.001
110° (mm)	29.0±3.2 (22.7~39.4)	31.5±5.2 (16.3~45.1)	38.2±9.8 (11.4~51.6)	<.001
120° (mm)	32.3±3.0 (27.0~42.7)	36.2±3.9 (28.7~46.5)	46.1±9.1 (32.4~78.0)	<.001
130° (mm)	33.6±2.6 (28.2~41.5)	37.0±3.2 (29.6~47.2)	44.4±5.4 (34.0~59.6)	<.001
P-value^b^	<.001	<.001	<.001	

MTA: maximum transverse drill angle without coming into contact with the medial condyle cartilage.

The lengths of tunnels (mm) are given as mean ± standard deviation (range).

^a^P-value for comparison of tunnel lengths between different transverse drill angles of MTA to MTA-20° in condition of fixed flexion angle.

^b^P-value for comparison of tunnel lengths between different flexion angles of 100° to 130° in condition of fixed transverse drill angle.

**Table 2:** Comparison of the proportions of short tunnel with less than 25 mm in each setting.

Flexion angle	Transverse drill angle			
	MTA	MTA-10°	MTA-20°	P-value^a^
100°	19 (63.3%)	27 (90%)	30 (100%)	<.001
110°	3 (10%)	2 (6.7%)	2 (6.7%)	0.779
120°	0 (0%)	0 (0%)	0 (0%)	-
130°	0 (0%)	0 (0%)	0 (0%)	-
P-value^b^	<.001	<.001	<.001	

MTA: maximum transverse drill angle without coming into contact with the medial condyle cartilage.

The values are given as cases with proportion in parentheses.

^a^P-value for comparison between different transverse drill angles of MTA to MTA-20° in condition of fixed flexion angle.

^b^P-value for comparison between different flexion angles of 100° to 130° in condition of fixed transverse drill angle.

**Table 3:** Comparison of the proportions of femoral tunnel wall breakage in each setting.

Flexion angle	Transverse drill angle			
	MTA	MTA-10°	MTA-20°	P-value^a^
100°	13 (43.3%)	20 (66.7%)	30 (100%)	<.001
110°	4 (13.3%)	9 (30.0%)	26 (86.7%)	<.001
120°	0 (0%)	0 (0%)	7 (23.3%)	0.001
130°	0 (0%)	0 (0%)	0 (0%)	-
P-value^b^	<.001	<.001	<.001	

MTA: maximum transverse drill angle without coming into contact with the medial condyle cartilage.

The values are given as cases with proportion in parentheses.

^a^P-value for comparison between different transverse drill angles of MTA to MTA-20° in condition of fixed flexion angle.

^b^P-value for comparison between different flexion angles of 100° to 130° in condition of fixed transverse drill angle.

**Table 4:** Comparison of mean graft bending angles at femoral tunnel aperture in each condition according to the flexion angle and transverse drill angle.

Flexion angle	Transverse drill angle			
	MTA	MTA-10°	MTA-20°	P-value^a^
100° (degree)	65.4±4.5 (59.1~71.3)	60.5±4.6 (53.9~66.4)	56.4±4.5 (49.8~62.1)	<.001
110° (degree)	72.5±4.8 (65.5~77.7)	69.0±4.4 (62.3~74.0)	66.2±4.0 (60.0~70.8)	<.001
120° (degree)	79.5±3.2 (75.6~83.5)	76.9±3.0 (72.9~80.6)	74.7±2.9 (70.7~78.2)	<.001
130° (degree)	84.8±4.7 (78.2~90.5)	83.8±4.5 (77.2~89.0)	83.0±4.2 (76.6~87.5)	0.007
P-value^b^	<.001	<.001	<.001	

MTA: maximum transverse drill angle without coming into contact with the medial condyle cartilage.

The graft bending angles (mm) are given as mean ± standard deviation (range).

^a^P-value for comparison of tunnel lengths between different transverse drill angles of MTA to MTA-20° in condition of fixed flexion angle.

^b^P-value for comparison of tunnel lengths between different flexion angles of 100° to 130° in condition of fixed transverse drill angle.

**Table 5:** Demographic data for patients.

Variable	(*n* = 30)
Sex^b^	
Male	17 (56.7%)
Female	13 (43.3%)
Age (years)^a^	40.3±16.8
Side^b^	
Right	13 (43.3%)
Left	17 (56.7%)
Transepicondylar distance (cm)^a^	82.1±5.7
Height (cm)^a^	168±8.6
Weight (kg)^a^	62.8±15.4

^a^The values are given as mean ± standard deviation.

^b^The values are given as *n* (%).

**Table 6:** Post hoc analysis of tunnel lengths between different flexion angles of 100° to 130° in condition of fixed transverse drill angle.

Pairwise comparison	MTA	MTA-10°	MTA-20°
100° versus 110°	<.001	<.001	<.001
100° versus 120°	<.001	<.001	<.001
100° versus 130°	<.001	<.001	<.001
110° versus 120°	<.001	<.001	0.015
110° versus 130°	<.001	<.001	0.013
120° versus 130°	<.001	0.158	0.516

MTA: maximum transverse drill angle without coming into contact with the medial condyle cartilage.

The values are given as adjusted P-value with use of Bonferroni correction.

**Table 7:** Post hoc analysis of tunnel lengths between different transverse drill angles of MTA to MTA-20° in condition of fixed flexion angle.

Pairwise comparison	100°	110°	120°	130°
MTA versus MTA-10°	<.001	<.001	<.001	<.001
MTA versus MTA-20°	<.001	<.001	<.001	<.001
MTA-10° versus MTA-20°	<.001	<.001	<.001	<.001

MTA: maximum transverse drill angle without coming into contact with the medial condyle cartilage.

The values are given as adjusted P-value with use of Bonferroni correction.

**Table 8:** The proportion of femoral tunnel wall breakage including entrance breakage and mid-tunnel breakage in each setting.

Flexion angle	Transverse drill angle								
	MTA			MTA-10°			MTA-20°		
	EB	MB	TB	EB	MB	TB	EB	MB	TB
100°	11 (36.7%)	2 (6.7%)	13 (43.3%)	9 (30%)	11 (36.7%)	20 (66.7%)	11 (36.7%)	19 (63.3%)	30 (100%)
110°	4 (13.3%)	0 (0%)	4 (13.3%)	7 (23.3%)	2 (6.7%)	9 (30.0%)	11 (36.7%)	15 (50%)	26 (86.7%)
120°	0 (0%)	0 (0%)	0 (0%)	0 (0%)	0 (0%)	0 (0%)	5 (16.7%)	2 (6.7%)	7 (23.3%)
130°	0 (0%)	0 (0%)	0 (0%)	0 (0%)	0 (0%)	0 (0%)	0 (0%)	0 (0%)	0 (0%)

MTA: maximum transverse drill angle without coming into contact with the medial condyle cartilage.

EB: entrance breakage, MB: mid-tunnel breakage, TB: total of femoral tunnel wall breakage including both entrance and mid-tunnel breakage.

The values are given as cases with proportion in parentheses.

**Table 9:** Post hoc analysis of graft bending angles at femoral tunnel aperture between different flexion angles of 100° to 130° in condition of fixed transverse drill angle.

Pairwise comparison	MTA	MTA-10°	MTA-20°
100° versus 110°	0.003	0.001	<.001
100° versus 120°	0.001	0.001	0.001
100° versus 130°	<.001	<.001	<.001
110° versus 120°	0.014	0.008	0.004
110° versus 130°	0.001	<.001	<.001
120° versus 130°	0.012	0.004	0002

MTA: maximum transverse drill angle without coming into contact with the medial condyle cartilage.

The values are given as adjusted P-value with use of Bonferroni correction.

**Table 10:** Post hoc analysis of graft bending angles at femoral tunnel aperture between different transverse drill angles of MTA to MTA-20° in condition of fixed flexion angle.

Pairwise comparison	100°	110°	120°	130°
MTA versus MTA-10°	<.001	<.001	<.001	0.010
MTA versus MTA-20°	<.001	0.001	<.001	0.023
MTA-10° versus MTA-20°	<.001	0.001	<.001	0.049

MTA: maximum transverse drill angle without coming into contact with the medial condyle cartilage.

The values are given as adjusted P-value with use of Bonferroni correction.

## Data Availability

The data used to support the findings of this study are available from the corresponding author upon request.
